# The osseointegration and stability of dental implants with different surface treatments in animal models: a network meta-analysis

**DOI:** 10.1038/s41598-021-93307-4

**Published:** 2021-07-05

**Authors:** Chun-Ping Hao, Nan-Jue Cao, Yu-He Zhu, Wei Wang

**Affiliations:** 1 Liaoning Provincial Key Laboratory of Oral Diseases, School and Hospital of Stomatology, China Medical University, Shenyang, Liaoning People’s Republic of China; 2 General Hospital of Northern Theater Command, Shenyang, Liaoning People’s Republic of China; 3 The Fourth Affiliated Hospital, Zhejiang University School of Medicine, Yiwu, Zhejiang People’s Republic of China

**Keywords:** Implants, Oral diseases

## Abstract

Dental implants are commonly used to repair missing teeth. The implant surface plays a critical role in promoting osseointegration and implant success. However, little information is available about which implant surface treatment technology best promotes osseointegration and implant stability. The aim of this network meta-analysis was to evaluate the osseointegration and stability of four commonly used dental implants (SLA, SLActive, TiUnite, and Osseotite). The protocol of the current meta-analysis is registered in PROSPERO (International Prospective Register of Systematic Reviews) under the code CRD42020190907 (https://www.crd.york.ac.uk). We conducted a systematic review following PRISMA and Cochrane Recommendations. Medline (PubMed), Cochrane Library, Embase, and the Web of Science databases were searched. Only randomized controlled trials were considered. Twelve studies were included in the current network meta-analysis, eleven studies were included concerning the osseointegration effect and five studies were included for stability analysis (four studies were used to assess both stability and osseointegration). Rank possibility shows that the SLActive surface best promoted bone formation at an early healing stage and TiUnite seemed to be the best surface for overall osseointegration. For stability, TiUnite seemed to be the best surface. The present network meta-analysis showed that the SLActive surface has the potential to promote osseointegration at an early stage. The TiUnite surface had the best effect on osseointegration regarding the overall healing period. The TiUnite surface also had the best effect in stability.

## Introduction

Oral implantology has thus far been the major choice for rehabilitation of edentulous regions. For the long-term success of dental implants, osseointegration is a prerequisite factor. Osseointegration means direct contact between the bone and the implant, which is often measured by the bone-to-implant contact (BIC) value under an optical microscope^1^.

The biocompatibility of implant surfaces is a critical promoter for osseointegration^1,2^. Surfaces with suitable roughness and high hydrophilicity are likely to promote more bone deposition than other surfaces. Currently, in clinical practice, there are four commonly used dental implants: SLA, SLActive, Osseotite, and TiUnite.

SLA has a rough surface that is produced by sandblasting with large grit followed by mixed acid etching with hydrochloric and sulfuric materials. Clinical trials demonstrate that the SLA surface could reduce the unloaded healing time from 12 to 6 weeks^3,4^. Currently, the SLA surface is the gold standard for developing novel implants. However, the sandblasting process of the SLA procedure may have blasting material embedded into the surface, hindering the osseointegration process. Moreover, the SLA surface is hydrophobic, which may disturb the initial cell attachment to implants^5^. To overcome these limitations, scientists have been devoted to successively modifying the properties of implant surfaces by improving surface wettability and optimizing surface chemistry.

The SLActive surface is an upgrade of the SLA surface so that it has higher wettability. It is prepared by rinsing SLA-treated implants under a nitrogen atmosphere and storing them in NaCl solution rather than placing them in dry storage^6^. Researchers have found that SLActive surfaces have favourable nano-roughness for bone deposition^7^. The SLActive surface could contribute to bone deposition around implants at an early stage and reduce healing time^8^. Reduced healing time could promote early functional loading, playing a critical role in increasing patients’ quality of life.

The Osseotite surface is another rough surface with a uniform micro-texture, which is produced by the dual acid (HCl-H_2_SO_4_) etching method. The acid etching method could roughen the implant surface without blasting material contamination. Many studies have suggested that Osseotite surfaces have achieved excellent outcomes under immediate loading conditions^9,10^.

TiUnite is a new-generation surface in the field of dental implants^11^. It is prepared by a specific oxidation process in which implants are treated in a galvanic cell containing phosphoric acid electrolyte. The TiUnite surface is characterized by a thick TiO_2_ layer enriched with highly crystalline calcium phosphate, which could promote apatite deposition around implants^12^.

The four dental implant surfaces have their own mechanisms in promoting bone deposition. It is unclear which implant is the most effective in promoting bone deposition. Moreover, there is little evidence-based support to guide implant choices in clinical practice.

In clinical practice, it is difficult to measure the osseointegration effect directly because the measurement of BIC value requires retrieving implants from patients. Clinicians typically use a resonance frequency analyser to measure the stability of dental implants to evaluate the osseointegration status indirectly. However, controversy exists about whether the implant stability quotient (ISQ) value could precisely indicate the osseointegration condition. Some studies found that there is a relationship between the ISQ and BIC value, while others showed that there is no significant relationship between the ISQ and BIC value^13–15^.

Therefore, we conducted a meta-analysis on animal models to directly compare the osseointegration effect of the four dental implant surfaces. We also synthesized evidence to further study the relationship between the ISQ value and BIC value.

Network meta-analysis is an extension of traditional meta-analysis. Network meta-analysis could synthesize direct and indirect evidence to enable an evaluation of the effect of multiple interventions at the same time^16^. Moreover, network meta-analysis provides higher precision by ranking all available treatments even though statistical analysis shows that there are no significant differences between interventions^17^.

In this network meta-analysis, we aimed to evaluate the osseointegration and stability of the above four commercially available implants in animal models. This network meta-analysis could provide support for clinical decision-making and point out directions for further research.

## Materials and methods

### Overview

The focused question of this review was to compare the effect of titanium implants with different surface treatments. The primary outcome was osseointegration measured by the BIC value. The second outcome was stability measured by ISQ values.

This network meta-analysis was conducted according to the PRISMA Extension Statement for Network Meta-analysis^18^. The questions were addressed with reference to participants or population (P); intervention (I); comparison, control, or comparator (C); outcome (O); and study design (S) (PICOS elements) (Table 1). The protocol of the current meta-analysis is registered in PROSPERO (International Prospective Register of Systematic Reviews) under the code CRD42020190907 (https://www.crd.york.ac.uk). Ethics approval was not required for this review.

**Table 1:** PICOS elements of the questions being addressed.

Participants	We will include studies researching the effect of different implant surfaces in animal models
Intervention and Comparison	We will include four implant surfaces: SLA, SLActive, Osseotite and TiUnite
Outcome	The primary outcomes of interest will be bone-to implant contact (BIC%). The second outcomes will be implant stability quotient (ISQ)
Study design	Random controlled experiment on animal models (all species, all genders)

### Search strategy and study selection

Medline (PubMed), Cochrane Library, Embase and the Web of Science databases were searched by two authors independently. The databases were searched up to June 2020. Only trials published in English were considered. For the Medline (PubMed) library, the searching strategy was as follows: (((((((dental implants[MeSH Terms]) OR (Dental implants[Title/Abstract])) OR (Implants, Dental[Title/Abstract])) OR (Dental Implant[Title/Abstract])) OR (Implant, Dental[Title/Abstract])) OR (Dental Prostheses, Surgical[Title/Abstract])) AND (((((((((Surface Properties[MeSH Terms]) OR (Surface Properties[Title/Abstract])) OR (Properties, Surface[Title/Abstract])) OR (Property, Surface[Title/Abstract])) OR (Surface Property[Title/Abstract])) OR (Wettability[MeSH Terms])) OR (Wettability[Title/Abstract])) OR (Implant surface treatment[Title/Abstract])) OR (Implant surfaces treatment[Title/Abstract]))) AND ((((((Osseointegration[MeSH Terms]) OR (Osseointegration[Title/Abstract])) OR (Peri-implant Endosseous Healing[Title/Abstract])) OR (Endosseous Healings, Peri-implant[Title/Abstract])) OR (Bone-to-implant contact[Title/Abstract])) OR (BIC[Title/Abstract]))AND(English[Language]).

For the electronic search, reference management software (Endnote X7) was used. Publications from Medline (PubMed), Cochrane Library, Embase, and Wed of Science databases were imported to this software, and duplicates were excluded. Two trained reviewers (Hao CP and Cao NJ) independently screened titles and abstracts for inclusion of potentially eligible trials. Then, full-text articles were accessed by the two reviewers independently to identify whether they could be included. Any disagreements were resolved by discussion, rereading and consultation with the last author (Wang W). The agreement between the two investigators was assessed by the kappa statistics.

**Figure 1:**
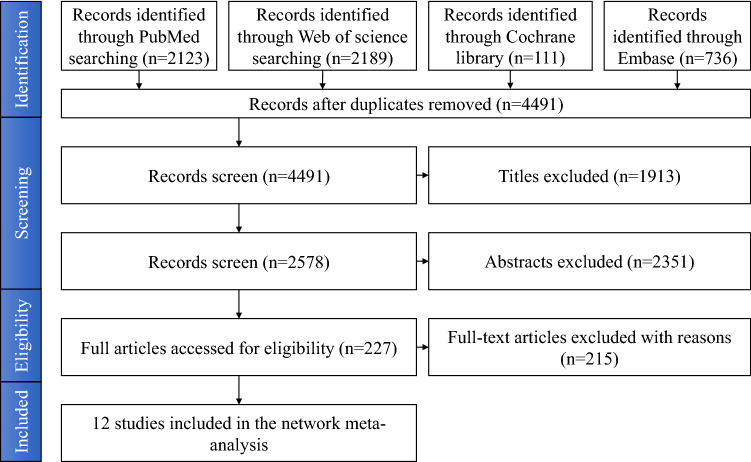
Prisma flowchart.

A flowchart presented in Fig. 1 shows the process of searching, screening, and determining eligibility according to the PRISMA Extension Statement for Network Meta-analyses^18^.

### Criteria for selecting studies for this review

Studies conducted in healthy animal models investigating the osseointegration and stability of titanium implants with SLA surfaces, SLActive surfaces, TiUnite surfaces, and Osseotite surfaces were included. Moreover, studies published in English were taken into account. Animal disease models, immediate implantation models, bone defect models, and studies on nontitanium implants were excluded.

### Data extraction

Data extraction from the included studies was conducted by two reviewers independently using a predesigned extraction form. The following key data were extracted from each included study: name of the first author, year of publication, sample size, mean and SD values of BIC and ISQ for each group and healing period. Studies providing all of this information were included in the current network meta-analysis. Where necessary, we contacted the authors of the included studies to collect missing data. Any disagreements were resolved by discussion with each other or consulting the third author (Zhu YH).

### Risk of bias

We assessed the quality of the included studies with SYRCLE’s risk of bias tool^19^. The following fronts were considered: selection bias (sequence generation, baseline characteristics, and allocation concealment), performance bias (random housing and blinding), detection bias (random outcome assessment and blinding), attrition bias (incomplete outcome data), reporting bias (selective outcome reporting), and other (other sources of bias).

Based on the characteristics of the current network meta-analysis, each animal model received different implants at the same time. Therefore, sequence generation, allocation concealment, random housing, blinding of performance bias (blinding trial caregivers and researchers), and random outcome assessment were not suitable for assessing the quality of studies included in our meta-analysis. The random placement of implants in animal models and random sacrifice of experimental animals at different time points may have influenced the outcome. Therefore, in the current network meta-analysis, we evaluated seven domains: baseline characteristics, random implant placement, blind outcome assessor, random sacrifice, incomplete outcome data, selective outcome reporting, and other sources of bias. Two independent authors (Hao CP and Cao NJ) assessed the studies and signified the risk of bias in the domains by indicating ‘yes’, ‘no’ or ‘uncertain’.

### Analysis

#### Statistical heterogeneity and meta-regression analysis

*The* Q-test and the I^2^ statistic were used to measure the heterogeneity of the network meta-analysis. According to the Cochrane recommendation, if I^2^ was below 40%, the heterogeneity could be considered low. If I^2^ was above 40%, we explored heterogeneity with network meta-regressions further. The potential source of heterogeneity could be the publication year, different animal species, healing period, and sample size.

#### Transitivity across treatment comparisons

Network meta-analysis is based on the transitivity assumption, which means that the distribution of potential effect modifiers is similar across various treatment comparisons^20^. The potential modifiers could be the year of publication, animal species, and sample size. We complied with the included criteria strictly to ensure that the included studies were sufficiently similar.

#### Inconsistency analysis

Node-splitting analysis was also conducted to identify statistical inconsistency in the network meta-analysis (NMA). There was no relevant inconsistency in the evidence when *P* > 0.05. Conclusions drawn from consistency models were reliable^21^. Nevertheless, if *P* < 0.05, we conducted sensitivity analyses to determine potential sources of inconsistency.

#### Publication bias

We conducted funnel plots to assess the publication bias in this meta-analysis. If the distribution was not roughly symmetrical, this was suggestive of an increased risk of bias^22^.

#### Grading of recommendations assessment, development, and evaluation

The evidence was evaluated by the CINeMA (Confidence in Network Meta-Analysis) system. CINeMA is a modified version of the GRADE (Grading of Recommendations, Assessment, Development, and Evaluations) approach, which grades evidence from network meta-analysis specifically^23^. We assessed the confidence of the results in six domains: within-study bias, indirectness, imprecision, heterogeneity, incoherence and publication bias. We evaluated each term by the quality of the included studies and the guidance of the CINeMA system.

#### Data synthesis and analysis

We used Stata 14 software and the st0410, st0411, and st0156-2 software packages to perform major data analysis^24,25^. We employed R software with the gemtc package to conduct meta-regression analysis^26^. As our results were extracted as continuous outcomes, the data are presented as the mean differences (MDs) with 95% confidence intervals. If the interventions showed no statistically significant difference, a ranking plot was conducted to explore the possible best measures^27^.

## Results

### Study selection

**Figure 2:**
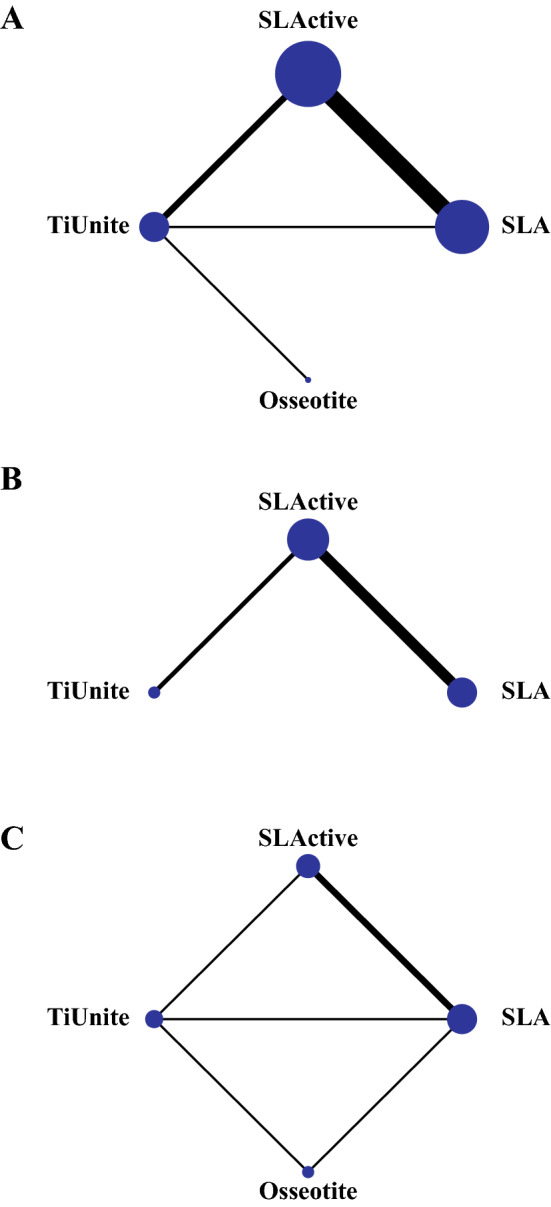
Network plot of comparisons. (**A**) BIC value of final healing stage; (**B**) BIC value of early healing stage; (**C**) ISQ value of final healing stage. The width of the lines is proportional to the number of trials directly comparing each pair of treatments. The size of each node is proportional to the number of studies for each intervention.

A PRISMA flow diagram describing the process of literature search and selection is presented in Fig. 1. The search of Medline (PubMed), Cochrane Library, Embase, and Web of Science databases revealed 5159 potentially relevant publications. After screening titles and abstracts, we selected 227 studies for further evaluation. A total of 215 articles were excluded after a full-text review due to their study design and no report of the outcome we were investigating. Therefore, 12 studies were included in the NMA. Their characteristics are described in Table 2. In addition, a network plot depicting the corresponding comparisons within the network is illustrated in Fig. 2. We assessed the agreement between the two investigators by Cohen’s kappa (ĸ) test. The kappa value was 0.83.

**Table 2:** The characteristics of included studies.

Author	Year	Study groups	Healing period	Country
Dagher	2014	Group 1: SLA (n = 4) Group 2: SLActive (n = 4) Group 3: TiUnite (n = 4)	2 Months	Lebanon
Lai	2009	Group 1: SLA (n = 2) Group 2: SLActive (n = 2)	8 Weeks	China
Rios-Santos	2018	Group 1: SLA (n = 4) Group 2: SLActive (n = 4)	8 Weeks	Spain
Streckbein	2013	Group 1: TiUnite (n = 6) Group 2: Osseotite (n = 6)	12 Weeks	Germany
Schlegel	2011	Group 1: SLA (n = 6) Group 2: SLActive (n = 6)	90 Days	Germany
Gottlow	2012	Group 1: SLActive (30) Group 2: TiUnite (30)	6 Weeks	Sweden
Abdel-Haq	2011	Group 1: SLA (n = 5) Group 2: SLActive (n = 5)	6 Weeks	Syria
Romero-Ruiz	2019	Group 1: SLA (n = 8) Group 2: SLActive (n = 8)	8 Weeks	Spain
Ernst	2014	Group 1: SLActive (n = 6) Group 2: TiUnite (n = 6)	8 Weeks	Switzerland
Choi	2018	Group 1: SLA (n = 4) Group 2: SLActive (n = 4)	10 Days	Korea
Buser	2004	Group 1: SLA (n = 7) Group 2: SLActive (n = 7)	8 Weeks	Switzerland
Sul	2009	Group 1: SLA (n = 10) Group 2: TiUnite (n = 10) Group 3: Osseotite (n = 10)	6 Weeks	Sweden

### Assessment of risk of bias

The quality of the included studies was assessed by two review authors independently using SYRCLE’s risk of bias tool. Regarding bias, we categorized studies as ‘yes’, ‘uncertain’ or ‘no’. The result showed overall good quality. Details of bias assessment are shown in Table 3. The support of bias judgement is presented in Supplementary Appendix S1.

**Table 3:** Risk of bias assessment of included studies.

Study	Baseline characteristics	Random implants placing	Blind outcome assessor	Random sacrifice	Incomplete outcome data	Selective outcome reporting	Other sources of bias
Dagher 2014	Low	High	Unclear	Unclear	Low	Low	Low
Lai 2009	Low	Low	Low	Unclear	Low	Low	Unclear
Rios-Santos 2018	Low	Low	Low	Low	Low	Low	Unclear
Abdel-Haq 2011	Low	Low	Low	Unclear	Low	Low	Low
Choi 2018	Low	Low	Unclear	Unclear	Low	Low	Low
Romero-Ruiz 2019	Low	Unclear	Unclear	Unclear	High	Low	Low
Schlegel 2011	Low	Low	Unclear	Unclear	Low	Low	Unclear
Gottlow 2012	Low	Low	Unclear	Unclear	Low	Low	Unclear
Ernst 2014	Low	Low	Unclear	Low	Low	Low	Unclear
Buser 2004	Low	Unclear	Unclear	Unclear	Low	Low	Unclear
Streckbein 2013	Low	Unclear	Unclear	Unclear	Low	Low	Unclear
Sul 2009	Low	Low	Unclear	Unclear	Low	Low	Low

### Osseointegration effect of implant surfaces

#### Osseointegration effect of implant surfaces in the overall healing period

A total of 11 studies^15,28–37^ with a sample size of 168 (40 implants with SLA surfaces, 76 implants with SLActive surfaces, 46 implants with TiUnite surfaces and 6 implants with Osseotite surfaces) were analysed for the effect of implant surfaces on osseointegration.

**Figure 3:**
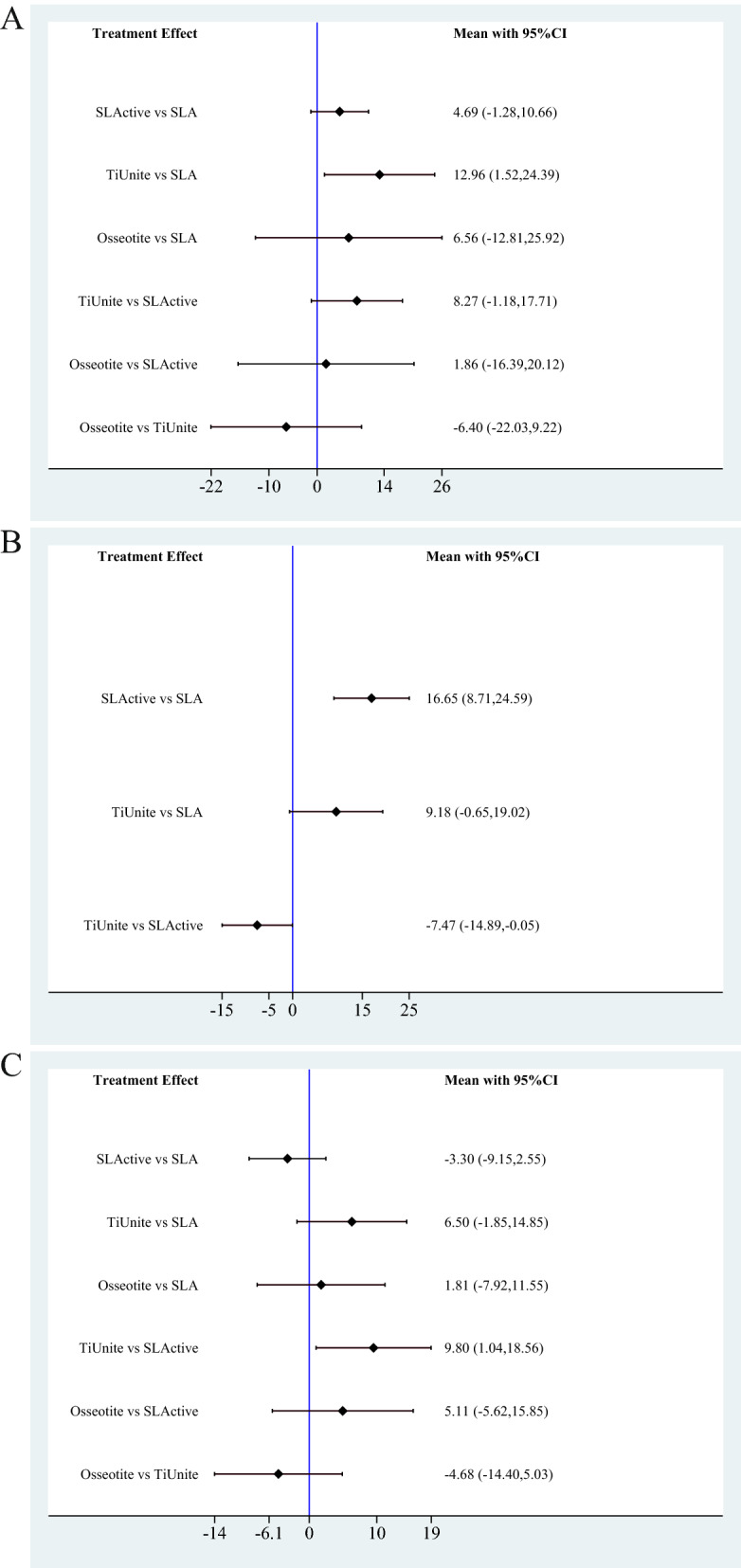
Forest plot of four titanium implant surfaces. (**A**) BIC value of final healing stage; (**B**) BIC value of early healing stage; (**C**) ISQ value of final healing stage.

The forest plot (Fig. 3A) showed that the TiUnite surface had a statistically significant advantage over the SLA surface (MD = 12.96; 95% CI 1.52, 24.39). There were no significant differences between other comparisons.

**Figure 4:**
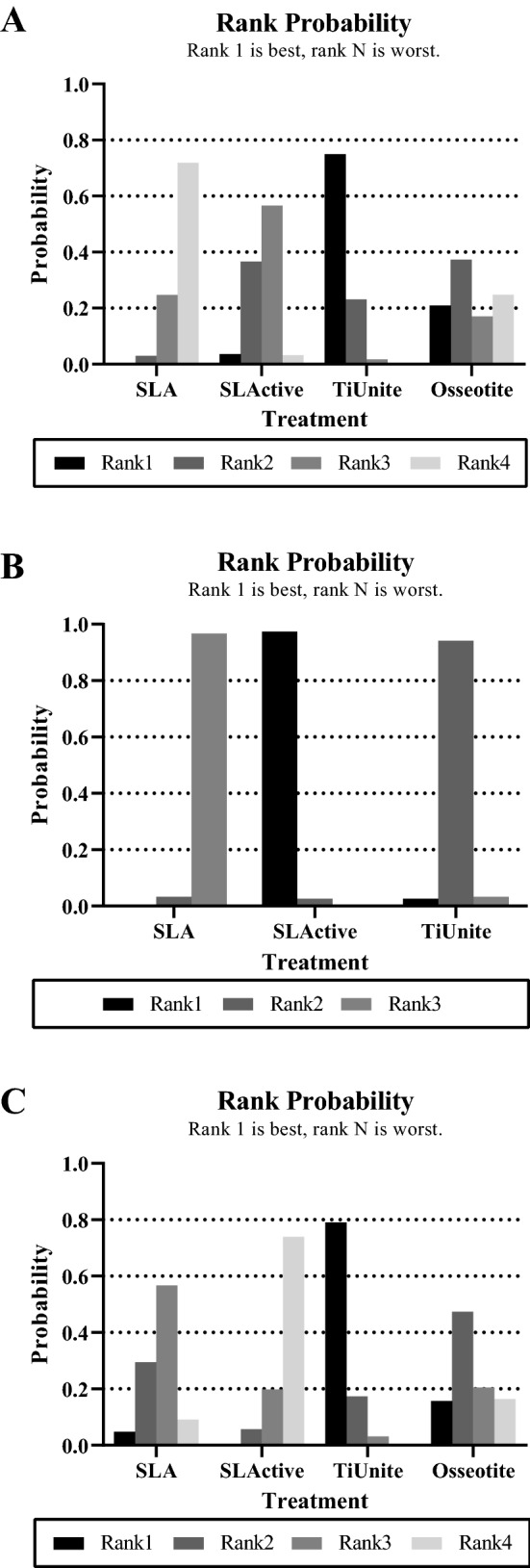
Rank probability plot of four titanium implant surfaces. (**A**) BIC value of final healing stage; (**B**) BIC value of early healing stage; (**C**) ISQ value of final healing stage.

Moreover, network meta-analysis could allow provide rank possibility calculations. If the effect size difference between treatments was small, it was possible to make decisions following the guidance of rank probabilities. The details of rank possibility are shown in Fig. 4A.

The rank plot (Fig. 4A) suggested that the TiUnite surface had the highest possibility of being the best surface to promote osseointegration (Rank 1 = 75.0), followed by the Osseotite surface (Rank 2 = 37.3), SLActive surface (Rank 3 = 56.6), and SLA surface (Rank 4 = 71.9).

#### Osseointegration effect of implant surfaces in the early healing period

To comprehensively investigate the osseointegration effect of implants, we further studied the osseointegration of implants at the early healing stage. Because studies researching Osseotite did not report BIC values at early stages, we only compared the osseointegration effects of SLA, SLActive, and TiUnite in the early healing period.

The forest plot (Fig. 3B) indicates that the SLActive surface had a statistical advantage over the SLA surface (MD = 16.65; 95% CI 8.71–24.59) and TiUnite surface (MD = 7.47; 95% 0.05–14.89). There was no significant difference between other comparisons. The rank plot revealed that SLActive had the highest possibility of being the most effective surface for osseointegration (Rank 1 = 97.4), followed by the TiUnite surface (Rank 2 = 94.1) and SLA surface (Rank 3 = 96.7) (Fig. 4B).

### Stability of implant surfaces

A total of 5^15,29,30,33,38^ studies with a sample size of 89 (27 implants with SLA surfaces, 30 implants with SLActive surfaces, 22 implants with TiUnite surfaces, and 10 implants with Osseotite surfaces) were analysed for the effect of different implant surfaces on stability.

The forest plot (Fig. 3C) indicated that TiUnite showed a significant advantage in stability over SLActive (MD = 9.8; 95% CI 1.04–18.56). The rank possibilities (Fig. 4C) showed that the TiUnite surface may have been the best in stability (Rank 1 = 79.0), followed by the Osseotite surface (Rank 2 = 47.4), SLA surface (Rank 3 = 56.7), and SLActive surface (Rank 4 = 74.0).

### Node-splitting analysis to assess inconsistencies in network meta-analysis

To evaluate the robustness of the models, we conducted node-split analysis. Node-split analysis reveals a potential inconsistency by assessing whether direct and indirect comparisons on a particular node (the split node) are in agreement^21^.

In current analyses, the BIC value in the early healing period did not show any inconsistencies. However, in the overall healing period, few inconsistencies were detected in the comparison between SLActive and TiUnite. To further explore the source of inconsistencies, we conducted sensitivity analysis.

We excluded studies with high risk of bias^15,36^. After omitting one study with a high risk of bias^36^, inconsistencies disappeared. After omitting this study, SLActive showed a significantly higher BIC value than SLA. The rank possibility was similar to the previous outcome. This outcome indicates that although a few inconsistencies exist in the current NMA, the results are reliable. As for stability, an inconsistency could be detected. To further explore the source of the inconsistency, we also conducted sensitivity analysis. After omitting one study^38^, the inconsistency disappeared. The rank possibility was similar to the previous outcome.

### Heterogeneity and network meta-regressions

The I^2^ of the BIC value in the early healing stage was 0% and that in the final healing stage was 10%, indicating that heterogeneity in our meta-analysis was low and that there was a high level of confidence in the evidence NMA.

We conducted meta-regressions to further explore the effect of potential modifiers (publication year, healing period, animal species, and sample size) on the final healing period. The results showed that these modifiers did not have a significant effect on outcomes. The details of the network meta-regressions are shown in Supplementary Appendix S2.

### Publication bias

**Figure 5:**
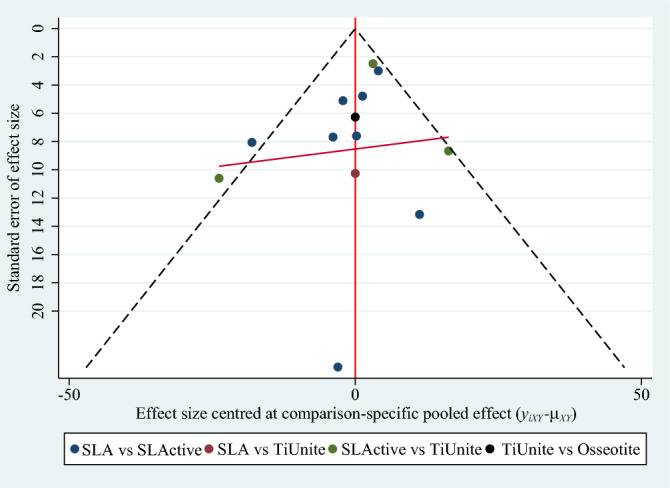
Funnel plot assessing publication bias of the final healing stage of BIC value.

We conducted funnel plots to assess publication bias in this meta-analysis. Visual inspection suggested that publication bias could be considered low (Fig. 5).

### Assessment of the confidence of evidence

The confidence of evidence was assessed using the CINeMA system. The comparisons in this network meta-analysis showed moderate and low rates of confidence. The details of the confidence of evidence are provided in Supplementary Appendix S3.

## Discussion

This network meta-analysis evaluates the osseointegration effect and stability of four implant surfaces (SLA, SLActive, Osseotite, and TiUnite). To the best of our knowledge, this network meta-analysis is the first to evaluate the effect of dental implant surfaces on the osseointegration effect and stability.

Compared with traditional meta-analyses, network meta-analyses could assess direct and indirect evidence and compare the effects of interventions^16^. Moreover, if the effect size between treatments was small, network meta-analysis provided the opportunity to determine rank possibilities of compared interventions to improve the accuracy of the assessment^17^.

For the overall osseointegration effect, eleven studies were included. Nine out of the 11 studies were conducted between 2010 and 2020, indicating that this problem is gaining attention. The rank possibility (Fig. 4A) showed that TiUnite may be the best surface, followed by Osseotite, SLActive, and SLA. Moreover, the forest plot (Fig. 3A) indicated that TiUnite had a significantly higher BIC value than SLA. There was no significant difference in the other comparisons.

Because the ability to promote early bone deposition is also important, we compared the osseointegration effect at the early healing period (approximately 2 weeks). The results revealed that SLActive had the highest osseointegration effect at the early stage. Although SLActive did not show a significant advantage over SLA in the overall healing period, SLActive had a significantly higher BIC value than SLA in the early healing period. Rapid osseointegration and shortened healing time are the goals of modern dentistry; these goals play critical roles in improving patients’ quality of life. Therefore, SLActive is an advanced surface too.

The reason that TiUnite showed the best osseointegration effect among the above four investigated surfaces in the overall healing period may be related to its advanced surface properties. The TiUnite surface is prepared by a special oxidation process to create a thick porous TiO_2_ layer^39^. The porous TiO_2_ layer is enriched with highly crystalline calcium phosphate to imitate the natural bone environment for cell attachment, which may have the potential to promote apatite formation^39^. Many studies have been conducted to research the performance of TiUnite surfaces in clinical practice. These studies found that this novel surface exhibited a higher bone-to-implant value than the turned surface, achieving a longer survival rate^40^. Therefore, the TiUnite surface may have advanced osteoconductive properties to enhance osseointegration^12^.

Although the rank possibility shows that the Osseotite surface may be the second-best surface in osseointegration, there is no statistically significant difference between the Osseotite surface and any of the other three surfaces. One possible reason for this outcome may be that the number of Osseotite implants in our network meta-analysis was small, which may have resulted in potential bias.

The SLActive surface is the third best surface for promoting osseointegration in the overall healing period. The SLActive surface was prepared by the same sandblasting and acid etching process as the SLA surface and then rinsed under a nitrogen atmosphere and stored in NaCl solution^41^. Researchers have proven that SLActive surfaces have higher wettability than SLA surfaces, reducing the water contact angle from 139.9° with SLA to 0° with SLActive, which may be due to the reduction in contamination^42^. High surface energy could enhance the interaction between the bone and implants^43^. It is believed that the higher surface energy of SLActive could achieve more bone apposition than the SLA surface at an early stage; however, the difference decreased as the healing time extended^44^. In our network meta-analysis, we verified the above hypothesis that the SLActive surface showed a significantly higher BIC value than the SLA surface at the early healing period; however, there was no significant difference between the two surfaces in the overall healing period. Therefore, SLActive is a valuable surface for promoting early healing and early loading.

The SLA surface was launched in 1998 by the ITI dental implant system^45^. It is a landmark in the field of dental implants with superior advantages in osseointegration. Currently, it is an industry standard to test novel implant surfaces. In the meta-analysis, the other three surfaces showed a better osseointegration effect than the SLA surface, indicating that these novel surfaces are great developments for promoting osseointegration.

In clinical practice, clinicians usually measure the ISQ value of dental implants to assess the implant osseointegration condition indirectly. However, the ability of the ISQ value to reflect the actual bone healing condition is doubtful. The correlation between the ISQ value and BIC value is controversial. In the present network meta-analysis, for stability, 5 studies were included with a sample size of 89. The forest plot shows that TiUnite has a significant advantage over SLActive, which is not consistent with the osseointegration outcome. These conflicting outcomes indicate that implant stability may not precisely reflect the actual bone healing condition. One explanation for this outcome may be that stability is influenced by many factors, e.g., bone condition, the site of implantation, implant method, and the length of the implants^46^.

In the current network meta-analysis, we revealed that the TiUnite surface is a promising technology. The TiO_2_ layer of TiUnite is effective in enhancing bone deposition. SLActive is an implant with considerable clinical value in promoting early osseointegration. The higher wettability of the SLActive surface is helpful in early bone deposition.

The major strength of this network meta-analysis was that the four researched implants are all commercially available. Compared with implants produced by researchers themselves, commercial implants have great homogeneity, improving the reliability of the current meta-analysis.

The present network meta-analysis also has some limitations. First, all of the included studies were animal experiments. Osseointegration in humans is different from that in animal models. It is difficult to find an ideal animal model due to the considerable differences in bone characteristics between animals and humans^47^. Therefore, the extrapolation of current outcomes to actual human conditions should be interpreted with caution. Second, numerous animal models were used in the 12 included studies, including sheep, dogs, pigs, and rabbits. To address this confounding factor, we conducted meta-regression analysis. The results indicated that animal species did not significantly modify the overall outcome. We expect further research to better address this issue and provide reliable guidance for clinical practice.

In conclusion, this network meta-analysis evaluated the osseointegration effect and stability of SLA, SLActive, TiUnite, and Osseotite surfaces. For osseointegration, our network meta-analysis shows for the first time that for a long observation period, the TiUnite surface is the best for promoting osseointegration, followed by the Osseotite, SLActive and SLA surfaces. However, the SLActive surface has the greatest advantage in promoting rapid osseointegration at an early stage. Regarding stability, all implants have ISQ value greater than 60 indicating they are all eligible^48,49^. Moreover, rank possibilities show that TiUnite is the best, followed by Osseotite, SLA, and SLActive. More comprehensive studies are needed to verify our findings.

## Conclusions

Our network meta-analysis shows that in the long-term healing period, TiUnite is the best for osseointegration among the four implants evaluated. Moreover, in the early healing stage, SLActive is the surface that provides the best osseointegration effect. Regarding stability, all implants are eligible with ISQ value greater than 60. Rank possibilities show that TiUnite seems to be the best, followed by Osseotite, SLA, and SLActive.

## Supplementary Information

Supplementary Information 1.

Supplementary Information 2.

## Data Availability

All data generated or analysed during this study are included in this published article (and its Supplementary Information files).
