# Supplementary material for: The osseointegration and stability of dental implants with different surface treatments in animal models: a network meta-analysis
Source: Sci Rep. 2021 Jul 5;11:13849. doi: 10.1038/s41598-021-93307-4 (PMC8257659; doi:10.1038/s41598-021-93307-4)
Supplement: Supplementary file 2 — Supplementary Information 2. [file 41598_2021_93307_MOESM2_ESM.docx]

# The osseointegration and stability of dental implants with different surface treatments in animal models: a network meta-analysis

Chun-Ping Hao^1,2,†^, Nan-Jue Cao^3,†^, Yu-He Zhu^1^, Wei Wang^1,*^

^1^ School and Hospital of Stomatology, China Medical University, Liaoning Provincial Key Laboratory of Oral Diseases, Shenyang, Liaoning, Peoples R China

^2^ General Hospital of Northern Theater Command, Shenyang, Liaoning, Peoples R China

^3^ The Fourth Affiliated Hospital, Zhejiang University School of Medicine, Yiwu, Zhejiang, Peoples R China

^†^ These authors contributed equally to this work.

* Correspondence should be addressed to Wei Wang: [wwang75@cmu.edu.cn](mailto:wwang75@cmu.edu.cn)

Table content

**Supplementary Appendix S1: The supporting of risk of bias judgement of included studies3-14**

Supplementary Appendix S2: Meta regressions15-30

Supplementary Appendix S3: Grading the primary evidence of current network meta-analysis using CINeMA31-32

**Supplementary Appendix S1. The supporting of risk of bias judgement of included studies**

We accessed the quality of included studies according to SYRCLE’s risk of bias tool. The supporting of judgement was presented in Tables.

Dagher M 2014

| Bias | | Authors’ judgement | Support for judgement |
| --- | --- | --- | --- |
| Selection bias | Baseline characteristics | Low risk | Four different implants were inserted per mandible, 2 implants on each side. |
|  | Random implants placing | High risk | For ease of identification, Aesthetica implants with a Euroteknika surface and NobelActive implants with TiUnite surface were placed in the left side, whereas the 2 Straumann SP implants, with SLA and SLActive surface, were placed in the right side. |
| Detection bias | Blind outcome assessor | Unclear | No details |
|  | Random sacrifice | Unclear | No details |
| Attrition bias | Incomplete outcome data | Low risk | Outcome were reported completely |
| Reporting bias | Selective outcome reporting | Low risk | The study protocol was available and all of the studys’ pre-specified primary and secondary outcomes were reported in the current manuscript |
| Other | Other sources of bias | Low risk | The study was funded by a grant from the research council of Saint Joseph’s University # FMD 81. |

Lai H. C. 2009

| Bias | | Authors’ judgement | Support for judgement |
| --- | --- | --- | --- |
| Selection bias | Baseline characteristics | Low risk | Implants were inserted in each side of the mandible of each dog. |
|  | Random implants placing | Low risk | SLA and modSLA implants were randomly assigned to either side. |
| Detection bias | Blind outcome assessor | Low risk | One special investigator performed histomorphometrical analyses and she was masked to the experimental conditions. |
|  | Random sacrifice | Unclear | No details |
| Attrition bias | Incomplete outcome data | Low risk | No implants were lost during the study period. |
| Reporting bias | Selective outcome reporting | Low risk | The study protocol was available and all of the studys’ pre-specified primary and secondary outcomes were reported in the current manuscript |
| Other | Other sources of bias | Unclear risk | This study was jointly supported by ITI Foundation for the Promotion of Implantology, Basel, Switzerland (Grant no. 499-2007) and Shanghai Municipal Education Committee (Grant no. 08zz57). Experimental implants were kindly provided by Institut Straumann AG, Basel, Switzerland. We can not access if the study was free from influence of funders |

Rios-Santos J. V. 2018

| Bias | | Authors’ judgement | Support for judgement |
| --- | --- | --- | --- |
| Selection bias | Baseline characteristics | Low risk | After these 6 months of healing, eight implants (one of each group) were randomly placed in the maxilla of each pig, which had previously had all their teeth removed in the first surgical procedure. |
|  | Random implants placing | Low risk |  |
| Detection bias | Blind outcome assessor | Low risk | The histological study was carried out in such a way that the histopathologist in charge of the histomorphometric study was unaware of the sample type in each animal being assessed. |
|  | Random sacrifice | Low risk | The 12 minipigs were randomly divided into three groups, placing eight implants in the maxillas of each pig, one of each type. Each group was sacrificed at a different time point from the second surgery, being 2, 4, and 8 weeks, respectively. |
| Attrition bias | Incomplete outcome data | Low risk | No implants were lost during the study period. |
| Reporting bias | Selective outcome reporting | Low risk | The study protocol was available and all of the studys’ pre-specified primary and secondary outcomes were reported in the current manuscript |
| Other | Other sources of bias | Unclear risk | This project has been financially supported by two research contracts with SOADCO SL, Project Code 1162 of the Research Foundation of the University of Seville and Straumann, Project Code: 1183 with Working Group Code 0480. |

Abdel-Haq J. 2011

| Bias | | Authors’ judgement | Support for judgement |
| --- | --- | --- | --- |
| Selection bias | Baseline characteristics | Low risk | A total of 30 implants consisting of 15 SLA and 15 modSLA standard implants were placed in the left and right tibiae of three sheep. |
|  | Random implants placing | Low risk | The distribution of five SLA and five modSLA implants in the left and right tibiae was determined using a previously generated random order for each sheep. |
| Detection bias | Blind outcome assessor | Low risk | All measurements were performed by an unbiased observer unaware of the different implant types (M.K.). |
|  | Random sacrifice | Unclear | No details |
| Attrition bias | Incomplete outcome data | Low risk | Radiographic examination taken at the end of the healing period revealed no pathology around implants (Fig. 3). In the stage of sacrifice and block retrieval of the tibiae, all implants were clinically stable and osseointegrated. |
| Reporting bias | Selective outcome reporting | Low risk | The study protocol was available and all of the studys’ pre-specified primary and secondary outcomes were reported in the current manuscript |
| Other | Other sources of bias | Low risk | Funding: This study was supported by a grant from Istanbul University Research Fund (2530). |

Choi J. Y. 2018

| Bias | | Authors’ judgement | Support for judgement |
| --- | --- | --- | --- |
| Selection bias | Baseline characteristics | Low risk | Each rabbit received four implants, two of which were placed in each tibia. |
|  | Random implants placing | Low risk | Different implants were placed in the rabbit tibiae according to the split-plot design. |
| Detection bias | Blind outcome assessor | Unclear | No details |
|  | Random sacrifice | Unclear | No details |
| Attrition bias | Incomplete outcome data | Low risk | No implant failure-related complications were reported during the healing period. |
| Reporting bias | Selective outcome reporting | Low risk | The study protocol was available and all of the studys’ pre-specified primary and secondary outcomes were reported in the current manuscript |
| Other | Other sources of bias | Low risk | This work was supported by grant no. 02-2016-0005 from the Seoul National University Dental Hospital (SNUDH) Research Fund. |

Romero-Ruiz M. M. 2019

| Bias | | Authors’ judgement | Support for judgement |
| --- | --- | --- | --- |
| Selection bias | Baseline characteristics | Low risk | After a 6-month healing period, three implants were placed in the maxilla of each pig, one for each surface studied, using a semi-submerged technique and in accordance with the manufacturer’s instructions. |
|  | Random implants placing | Unclear | No details |
| Detection bias | Blind outcome assessor | Unclear | No details |
|  | Random sacrifice | Unclear | No details |
| Attrition bias | Incomplete outcome data | High risk | The statement of sample size is confusing. |
| Reporting bias | Selective outcome reporting | Low risk | The study protocol was available and all of the studys’ pre-specified primary and secondary outcomes were reported in the current manuscript |
| Other | Other sources of bias | Low risk | The study was founded by the Project Code 1871 Agreement/Contract type L.O.U. between SOADCO and the F.I.U.S. (Research Foundation of the University of Seville) (Dr. Ríos-Santos). |

Schlegel K. A. 2011

| Bias | | Authors’ judgement | Support for judgement |
| --- | --- | --- | --- |
| Selection bias | Baseline characteristics | Low risk | After the randomized placement of six implants per animal (each three SLA® and three SLActive® implants per animal; 96 implants in total) according to the standard protocol for the implant system used (Straumann Bone Level® implants, Straumann GmbH), the periosteum and skin were sutured in two layers. |
|  | Random implants placing | Low risk |  |
| Detection bias | Blind outcome assessor | Unclear | No details |
|  | Random sacrifice | Unclear | No details |
| Attrition bias | Incomplete outcome data | Low risk | The outcome of healthy model is reported completely. |
| Reporting bias | Selective outcome reporting | Low risk | The study protocol was available and all of the studys’ pre-specified primary and secondary outcomes were reported in the current manuscript |
| Other | Other sources of bias | Unclear | This study was funded by the ELAN of the University of Erlangen-Nuremberg (ELAN-55410013) and the ITI Foundation/Research Committee (ITI- 5052007). The authors state that there are no conflicts of interest. |

Gottlow J. 2012

| Bias | | Authors’ judgement | Support for judgement |
| --- | --- | --- | --- |
| Selection bias | Baseline characteristics | Low risk | Surgery was made under sterile conditions. The tibial methaphysis and the distal femoral condyle of both legs were used as experimental sites and assigned to test and control implants using a rotational scheme. |
|  | Random implants placing | Low risk |  |
| Detection bias | Blind outcome assessor | Unclear | No details |
|  | Random sacrifice | Unclear | No details |
| Attrition bias | Incomplete outcome data | Low risk | Outcome were reported completely. |
| Reporting bias | Selective outcome reporting | Low risk | The study protocol was available and all of the studys’ pre-specified primary and secondary outcomes were reported in the current manuscript |
| Other | Other sources of bias | Unclear | The study was supported by Institut Straumann AG, Basel, Switzerland. |

Ernst S. 2014

| Bias | | Authors’ judgement | Support for judgement |
| --- | --- | --- | --- |
| Selection bias | Baseline characteristics | Low risk | All dental implants were placed in the cranial part of the left (n = 6) and right (n = 6) pelvis of each animal, alternating on either side of the linea glutea of the iliac wing. |
|  | Random implants placing | Low risk |  |
| Detection bias | Blind outcome assessor | Unclear | No details |
|  | Random sacrifice | Low risk | Animals were randomly allocated to three time points of 2, 4 and 8 weeks. |
| Attrition bias | Incomplete outcome data | Low risk | Outcome were reported completely. |
| Reporting bias | Selective outcome reporting | Low risk | The study protocol was available and all of the studys’ pre-specified primary and secondary outcomes were reported in the current manuscript |
| Other | Other sources of bias | Unclear | Noble Biocare, Kloten, Switzerland is highly acknowledged for financially supporting this study (Grants 1198 and 1199). |

Buser D 2004

| Bias | | Authors’ judgement | Support for judgement |
| --- | --- | --- | --- |
| Selection bias | Baseline characteristics | Low risk | Depending on the anatomical situation, 3 or 4 implants were inserted on either side of the maxilla, in a split-mouth design. |
|  | Random implants placing | Unclear | No details |
| Detection bias | Blind outcome assessor | Unclear | No details |
|  | Random sacrifice | Unclear | No details |
| Attrition bias | Incomplete outcome data | Low risk | Outcome were reported completely. |
| Reporting bias | Selective outcome reporting | Low risk | The study protocol was available and all of the studys’ pre-specified primary and secondary outcomes were reported in the current manuscript |
| Other | Other sources of bias | Unclear | The study was funded by the ITI Foundation for the Promotion of Implantology, Basel, Switzerland (Grant No. 175/1999). |

Streckbein P. 2013

| Bias | | Authors’ judgement | Support for judgement |
| --- | --- | --- | --- |
| Selection bias | Baseline characteristics | Low risk | Four different implant types were placed on each side of the lower jaw, according to the respective manufacturer’s recommendations. |
|  | Random implants placing | Unclear | No details |
| Detection bias | Blind outcome assessor | Unclear | No details |
|  | Random sacrifice | Unclear | No details |
| Attrition bias | Incomplete outcome data | Low risk | The outcome interested were reported completely. |
| Reporting bias | Selective outcome reporting | Low risk | The study protocol was available and all of the studys’ pre-specified primary and secondary outcomes were reported in the current manuscript |
| Other | Other sources of bias | Unclear | This study was supported by Nobel Biocare Deutschland GmbH, Köln, Germany, 3i, Miami, FL, USA, Densply Friadent GmbH, Mannheim, Germany, and IGfZ eG, Diez, Germany |

Sul Y. T 2009

| Bias | | Authors’ judgement | Support for judgement |
| --- | --- | --- | --- |
| Selection bias | Baseline characteristics | Low risk | Six implants, one of each group, were randomly placed in the two tibiae of one rabbit. |
|  | Random implants placing | Low risk |  |
| Detection bias | Blind outcome assessor | Unclear | No details |
|  | Random sacrifice | Unclear | No details |
| Attrition bias | Incomplete outcome data | Low risk | The outcome interested were reported completely. |
| Reporting bias | Selective outcome reporting | Low risk | The study protocol was available and all of the studys’ pre-specified primary and secondary outcomes were reported in the current manuscript |
| Other | Other sources of bias | Low risk | We would like to acknowledge research grants from the Biotechnology Development Project (2007-04306) from the Ministry of Education & Human Resource Development, Republic of Korea, and the Swedish Medical Research Council. |

**Supplementary Appendix S2. Meta regressions**

We evaluated the effect of several covariates on primary outcome (BIC value in final healing stage): animal species, healing period, sample size, and publication year. None of covariates had significant effect on overall outcome. The following paragraphs list the effect of covariates on overall outcome.

**1. Species**

When assessing the influence of animal species on overall outcome, 95%CI crossing 0 means no statistical significance.

Regression coefficient of each treatment when regression on “animal species”

| Regression coefficient | 95%CI |
| --- | --- |
| Beta [SLActive] | (-23.39, 12.02) |
| Beta [TiUnite] | (-52.14, 27.17) |
| Beta [Osseotite] | (-122.90, 223.83) |


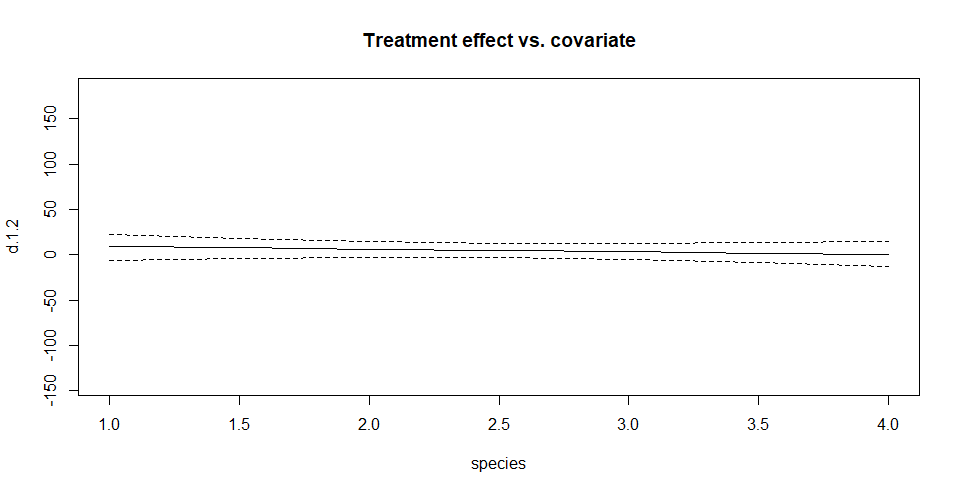

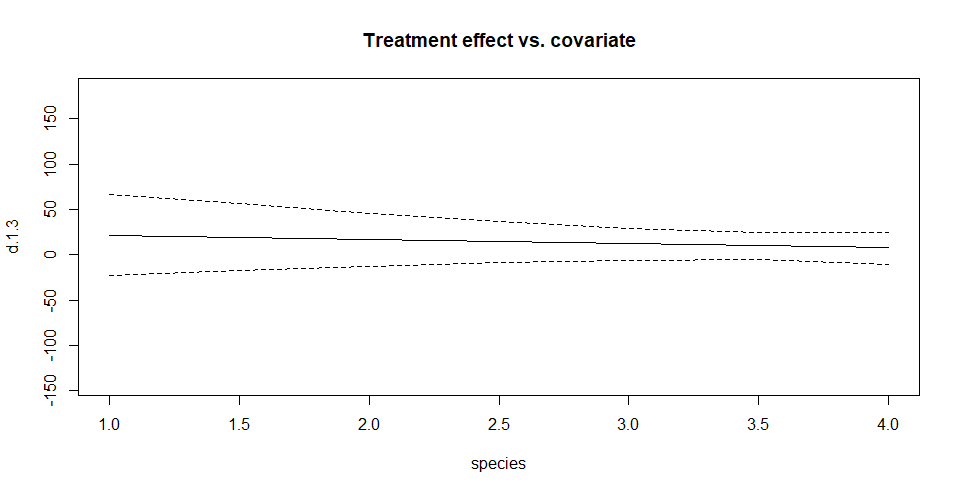

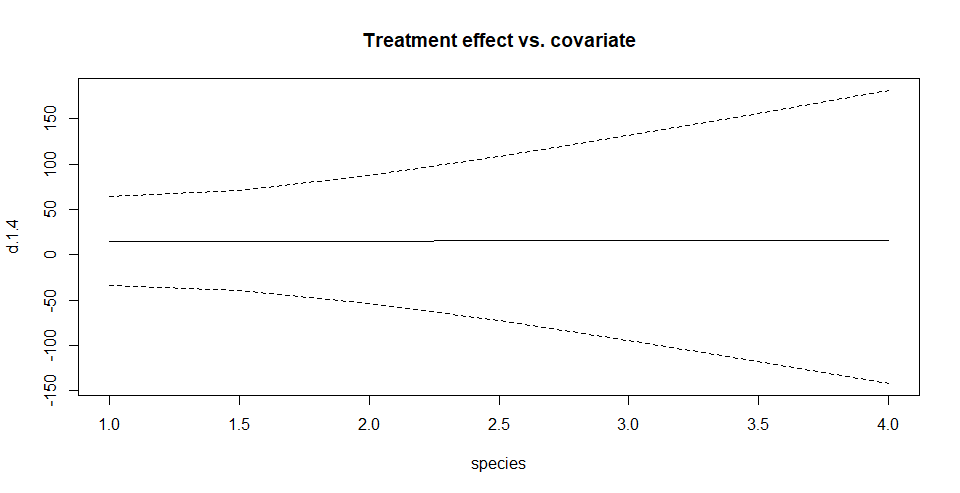


Figure 1: Covariate effect of animal model species. D.1.2, d.1.3, d.1.4 refer to the comparisons between SLA and SLActive,SLA and TiUnite, SLA and Osseotite respectively.

**2. Publication year**

Publication year does not have significant modifier effect on outcome too. 95%CI of regression coefficient crossing 0 means no statistical significance.

Regression coefficient of each treatment when regression on “publication year”

| Regression coefficient | 95%CI |
| --- | --- |
| Beta [SLActive] | (-8.49, 16.98) |
| Beta [TiUnite] | (-74.30, 49.08) |
| Beta [Osseotite] | (-278.31, 265.38) |


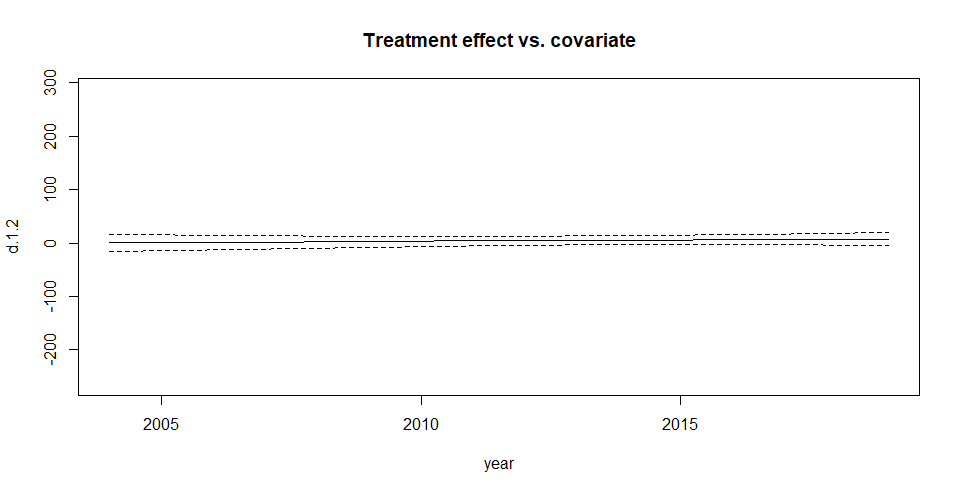

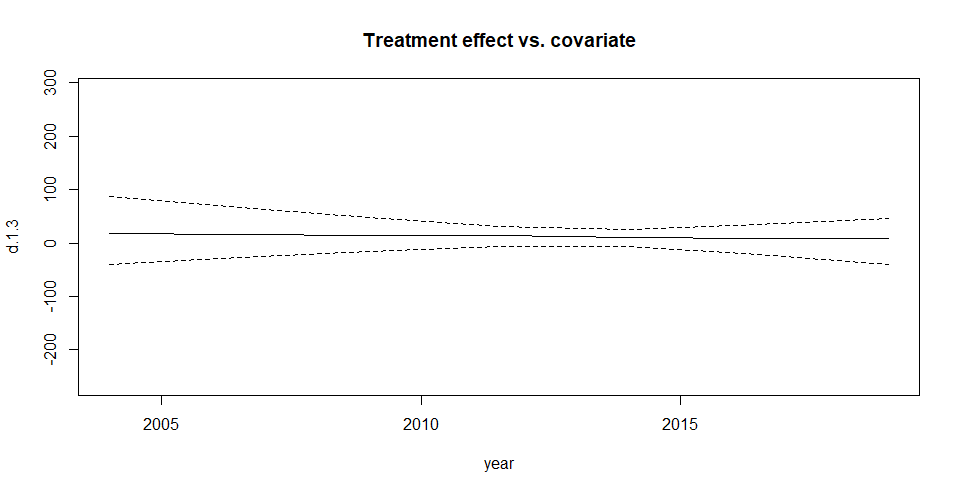

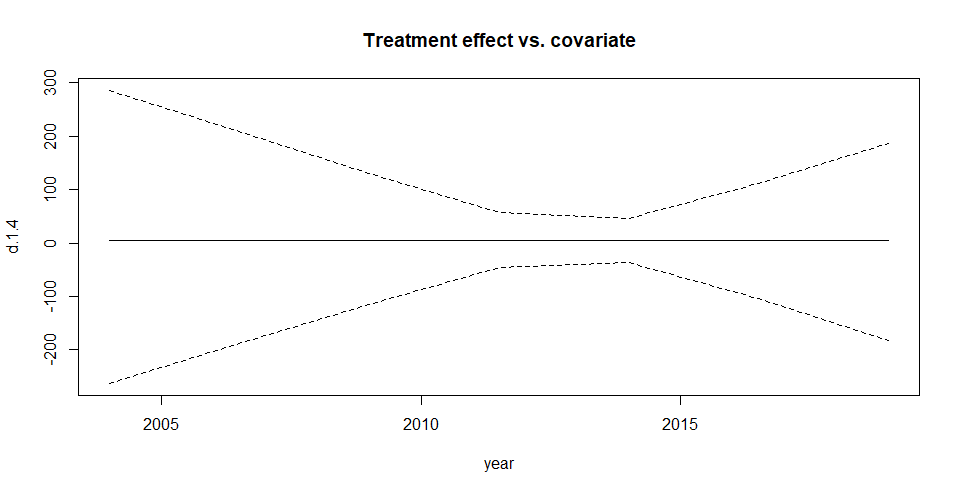


Figure 2: Publication year. D.1.2, d.1.3, d.1.4 refer to the comparisons between SLA and SLActive,SLA and TiUnite, SLA and Osseotite respectively.

**3. Sample Size**

Sample size does not have significant modifier effect on outcome. 95%CI of regression coefficient crossing 0 means no statistical significance.

Regression coefficient of each treatment when regression on “sample size”

| Regression coefficient | 95%CI |
| --- | --- |
| Beta [SLActive] | (-30.04, 37.41) |
| Beta [TiUnite] | (-25.55, 41.02) |
| Beta [Osseotite] | (-207.77, 304.53) |


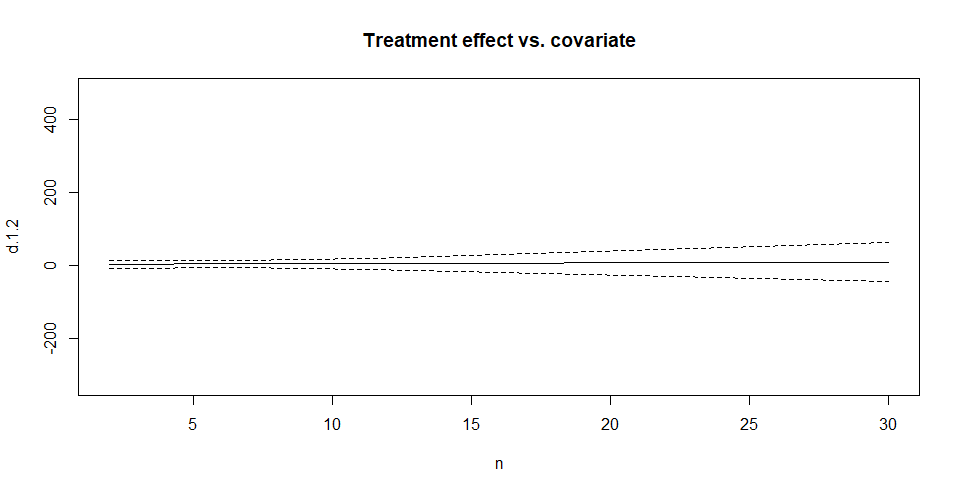

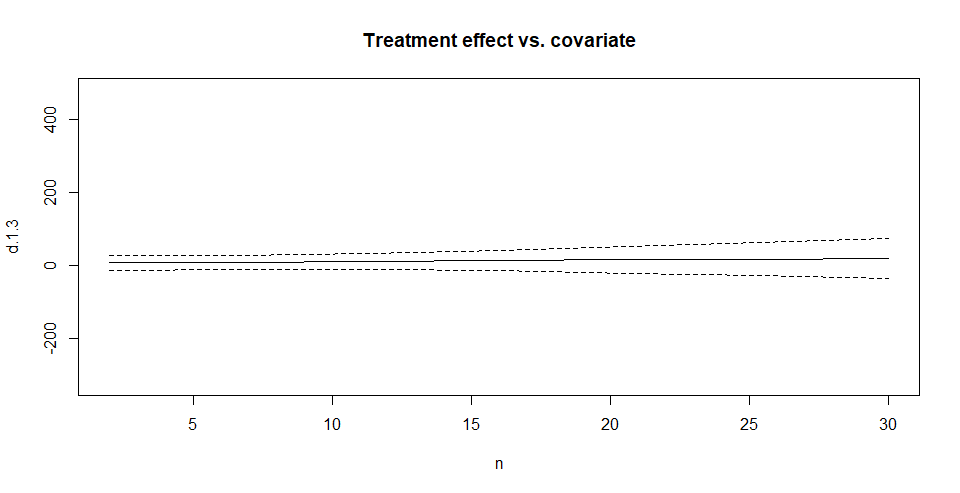

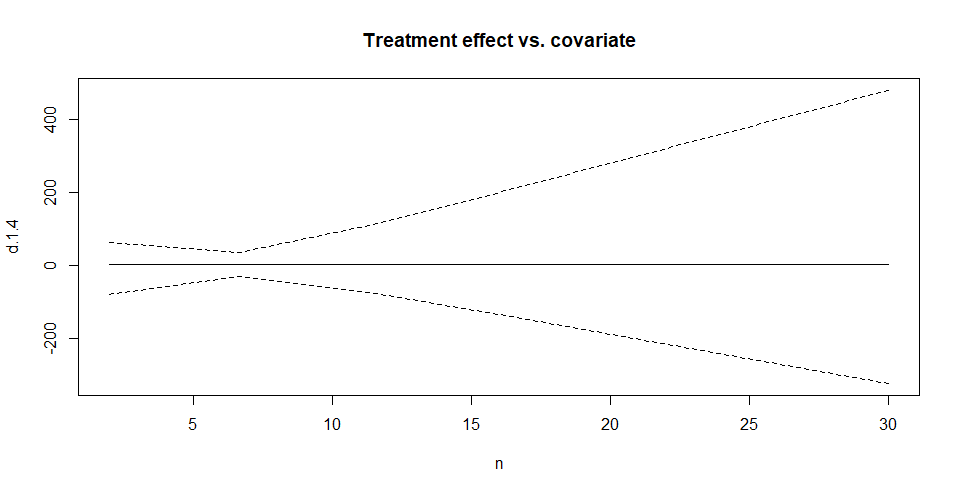


Figure 3: Sample Size. D.1.2, d.1.3, d.1.4 refer to the comparisons between SLA and SLActive, SLA and TiUnite, SLA and Osseotite respectively.

**4. Healing Period**

Healing period does not exert significant effect on outcome. 95%CI of regression coefficient crossing 0 means no statistical significance.

Regression coefficient of each treatment when regression on “healing period”

| Regression coefficient | 95%CI |
| --- | --- |
| Beta [SLActive] | (-20.68, 14.00) |
| Beta [TiUnite] | (-49.53, 35.74) |
| Beta [Osseotite] | (-277.44, 389.93) |


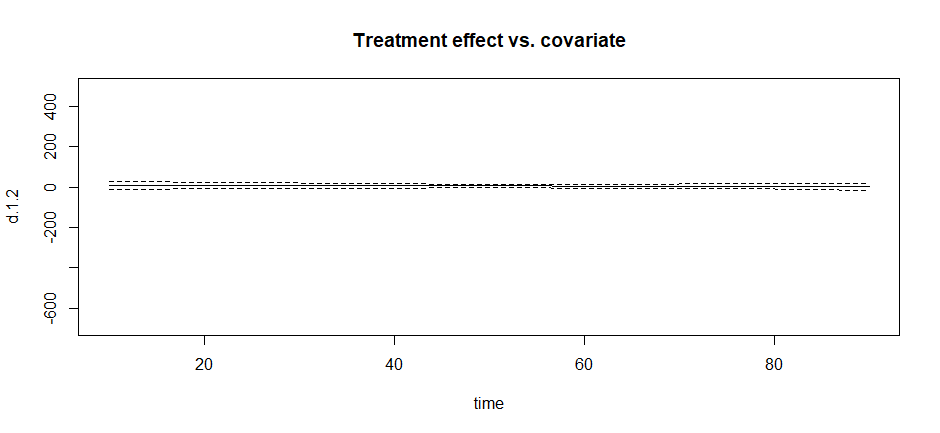

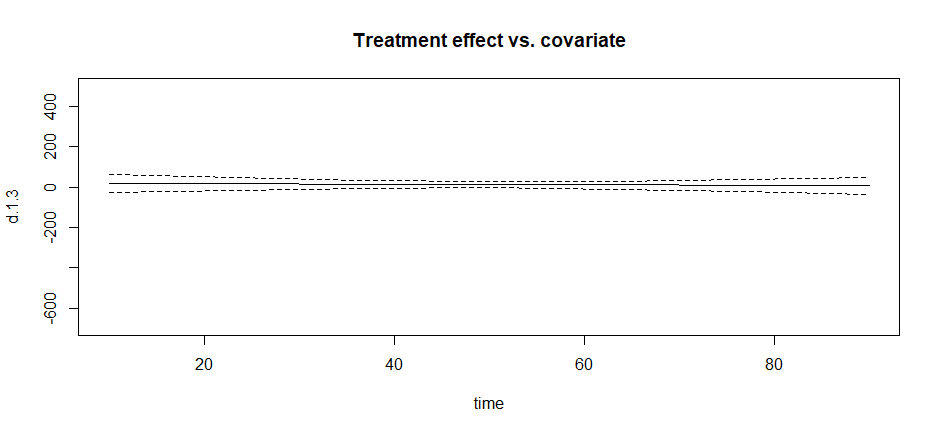

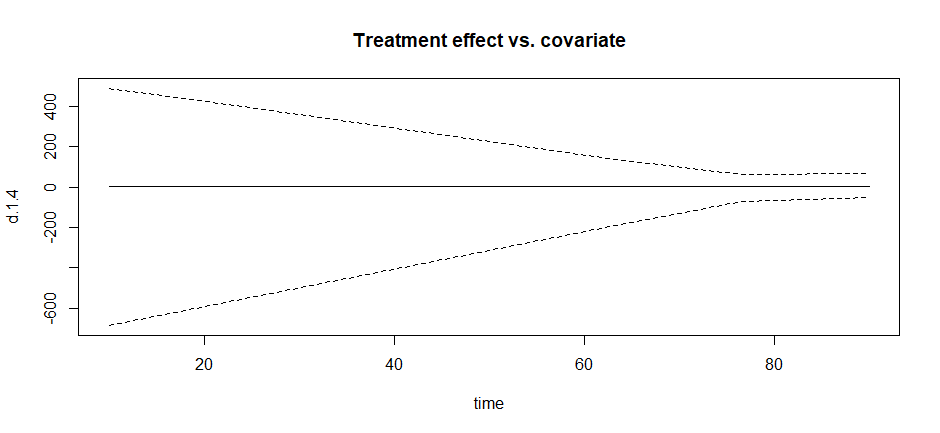


Figure 4: Healing period. D.1.2, d.1.3, d.1.4 refer to the comparisons between SLA and SLActive,SLA and TiUnite, SLA and Osseotite respectively.

**Supplementary Appendix S3. Grading the primary evidence of current network meta-analysis using CINeMA**

We judge the evidence of network meta-analysis according to the online documentation of CINeMA (http://cinema.ispm.ch/).

**1. Within-study bias**

The assessment of within-study bias is based on the quality of included studies. We have evaluated studies’ quality using SYRCLE’s risk of bias tool. The following table shows how the global rating is made based on the individual risk of bias items.

Global rating based on the individual risk of bias items.

| Global rating | Items with high risk | Items with unclear risk |
| --- | --- | --- |
| Low risk | 0 | =<3 |
| Moderate risk | 0 | >3 |
| Moderate risk | 1 | any |
| High risk | >1 | any |

**2. Across-study bias (Publication bias)**

Our search strategy is professional and complete including three databases, PubMed, Cochrane, and Embase, as recommended by Cochrane handbook. We also searched web of science to find as more studies as possible. As there are more than 10 studies on the BIC value at final healing stage, we also conducted a funnel plot to evaluate potential publication bias. The funnel plot is roughly symmetrical suggesting that the publication bias could be taken as low.

**3. Indirectness**

The included criterion of current meta-analysis is strict. Studies on disease models, poor bone quality, immediate loading, defect bone model, zirconia implants are excluded. Therefore, we did not downgrade evidence in indirectness.

**4. Imprecision**

Because our outcome is continuous, the meaningful threshold is set at a mean difference of higher or lower than 0. If the confidence interval crossed 0 the comparison was downgraded one level.

**5. Heterogeneity**

The clinically important size of effect is defined as mean difference of higher or lower than 0. The advice provided by CINeMA automatically was followed.

**6. Incoherence**

We assessed the global incoherence based on a random-effects design-by-treatment interaction model. Local Incoherence is tested by Separate Indirect from Direct Evidence. We adapt the judgement provided by CINeMA automatically.

The overall judgement of confidence in evidence for primary outcome: BIC value in final healing period.

| Comparison | Nature of evidence | Confidence level | Downgrading |
| --- | --- | --- | --- |
| SLA vs SLActive | mixed | moderate | imprecision |
| SLA vs TiUnite | mixed | moderate | heterogeneity |
| SLActive vs TiUnite | mixed | moderate | imprecision |
| TiUnite vs Osseotite | mixed | low | imprecision, incoherence |
| SLA vs Osseotite | indirect | low | imprecision, incoherence |
| SLActive vs Osseotite | indirect | low | imprecision, incoherence |
